# A discourse analysis of male sexuality in the magazine *Intimacy*

**DOI:** 10.4102/phcfm.v7i1.691

**Published:** 2015-03-19

**Authors:** Rory du Plessis

**Affiliations:** 1 Department of Visual Arts, University of Pretoria, South Africa

## Abstract

**Background:**

The World Health Organization's publication, *Developing sexual health programmes*, states that the media is an important source of information about sexuality. Although the media can promote awareness of sexual health issues, it also acts as a vehicle for defining and regulating sex norms. In other words, the standards of ‘normal’ sex are in part defined by the media. Accordingly, it has become imperative to analyse the media's construction of sexual norms in order to reveal how they are related to specific ideological views. For the purposes of this study, the focus will be limited to analysing the South African publication *Intimacy*.

**Aim:**

The study aims to reveal how the sex advice articles written in *Intimacy* for women in regard to their male partner's sexuality reflect patriarchal and phallocentric ideologies.

**Method:**

A discourse analysis of the sex advice articles in the magazine *Intimacy* was conducted. It was informed by feminist theories of sexuality that seek to examine the ways in which texts are associated with male-centred versions of sexual pleasure.

**Results:**

The discourse analysis identified a number of key themes regarding male sexuality. These include: (1) biological accounts of male sexuality; (2) phallocentric scripting of the sex act; and (3) the melodramatic penis.

**Conclusion:**

Constructions of male sexuality require the inclusion of alternative modes of male erotic pleasure. This requires texts that encourage men to explore and also to experiment with pleasurable feelings associated with non-genital erogenous zones of the body.

## Abstrait

**Contexte:**

La publication de l’Organisation mondiale de la Santé, *Développement de programmes de santé sexuelle*, déclare que les médias sont une source importante d’informations sur la sexualité. Bien que les médias sensibilisent les gens aux problèmes de santé sexuelle, ils servent aussi de véhicule pour définir et réglementer les normes sexuelles. Autrement dit, les normes sexuelles ‘normales’ sont en partie définies par les médias. Ainsi, il est donc impératif d’analyser la construction des normes sexuelles des médias pour montrer leur corrélation par rapport à des points de vue idéologiques. Pour les besoins de cette étude, nous nous limiterons à l’analyse de la revue sud-africaine *Intimacy*.

**Objet:**

L’étude a pour but de montrer comment les articles du magazine *Intimacy* qui donnent des conseils sur la sexualité aux femmes à l’égard de la sexualité de leur partenaire masculin reflètent des idéologies patriarcales et phallocentriques.

**Méthode:**

Une analyse a été faite du discours des articles qui donnent des conseils sur la sexualité dans le magazine *Intimacy.* Il est influencé par les théories féministes sur la sexualité qui cherchent à examiner de quelle manière les textes sont centrés sur le plaisir sexuel des hommes.

**Résultats:**

L’analyse du discours a identifié un certain nombre de thèmes clés concernant la sexualité masculine. Ce sont: (1) les récits naturalistes biologiques de la sexualité masculine; (2) les scénarios phallocentriques de l’acte sexuel; et (3) le pénis mélodramatique.

**Conclusion:**

Les construits de la sexualité masculine requièrent l’inclusion de modes alternatifs de plaisir érotique masculin. Cela demande des textes qui encouragent les hommes à explorer et aussi à expérimenter des sentiments agréables associés aux zones érogènes non génitales du corps.

## Introduction

The World Health Organization's publication, *Developing sexual health programmes*, states that the media is an important source of information about sexuality.^1^ Although the media can promote awareness of sexual health issues, it also acts as a vehicle for producing normative notions of sex and, consequently, it plays a role in regulating current trends in sexual practices.^2^ It is precisely in this role of being a major vehicle for the display and explanation of sexuality that the media has come to replace religious and moral leaders as sexual authorities in the public's pursuit of sexual ‘normalcy’.^3^ In other words, the standards of ‘normal’ sex are, in part, defined by the media.^2^,^3^ Accordingly, it has become imperative to analyse the media's construction of sexual norms in order to reveal how they are related to specific ideological views.^3^ Recent South African scholarship has focused specifically on investigating the role of magazines and print media in the construction and dissemination of normative notions of sex.^4,5,6^ This study endeavours to continue in a similar vein as such scholarship by analysing the South African magazine publication, *Intimacy*.

The publication in question endeavours to provide its readers with information for a healthy, sensual and passionate lifestyle (Fernandez J 2008, personal communication, August 27). In particular, it intends to empower women to take control of their sex life.^7^ This is underscored in the publication discussing ‘all intimate and health issues woman face daily – How to put the passion back in your relationship, contraceptives, pregnancy, infertility, Breast cancer, Menopause, low libido, cervical cancer, etc.’ (Fernandez J 2008, personal communication, August 27). As will be shown in the analysis that follows, such discussions which embody the hallmarks of female sexual empowerment^8^ are obstructed by descriptions of male sexuality, found within the same publication, that both accept and reinforce dominant gender and sex norms. To elucidate further, although the magazine celebrates the right of women to desire sex and experience sexual pleasure, the sex advice written in *Intimacy* for women relating to their male partner's sexuality is limited to male-centred sex acts and sexual practices that are based on traditional gender roles, ideals and expectations.^8^

To put it succinctly, the study aims to reveal how the sex advice articles written for women regarding male sexuality reflect patriarchal and phallocentric ideologies. These ideologies ensure that sexuality is male-centred, which results in the precedence of male sexual needs. The key themes that contribute to the aforementioned ideologies include:

- Patriarchal: female submission to the sexual needs of men whilst at the same time providing emotional support to them. In other words, women are tasked with not only pleasing a man sexually but also caring for his self-esteem.^8,9,10^
- Phallocentric: penile erections are viewed as being the essence of male sexuality and satisfaction. Furthermore, ‘real’ sex is limited and valorised to a coital scenario – the penetration of the vagina by the penis.^2,3,8,11^

Such ideological underpinnings perpetuate narrow ideas of sex, sexuality and gender relations whilst delimiting the sexual act, female sexuality and male sexuality to predefined potentials and gender relations.^2^ In view of this, the study takes a ‘sex critical’^12^ approach that seeks to analyse all forms of sexuality and sexual practices in order to reveal the presence of any normative values that perpetuate and uphold patriarchal ideologies.^12^

The research methodology of this study is a discursive analysis of the text in question. Discourse analysis does not seek to validate the ‘truth’ about male and female sexuality in this particular case but rather reveals the ways in which these ‘truths’ and our knowledge of sexuality are structured by ideology.^2,13^ Accordingly, discourse analysis problematises purely biological accounts of human sexuality and instead argues that our understanding and interpretation of sexuality are shaped by ideological, socio-cultural and historical influences. As such, a discourse analysis of the given text aims to reveal the ways in which ideologies are embodied, manifested and reflected in the discussion of sexual acts (how the various sex acts are defined, classified and promoted), as well as the ways in which the sexual acts are connected to governing the conduct and relations between men and women.

In this discourse analysis, a number of key themes regarding male sexuality which reflect a patriarchal and phallocentric agenda are identified. These are: (1) biological accounts of male sexuality: that the ‘needs’ of the male sex drive dictate the sexual encounter; (2) phallocentric scripting of the sex act: male sexuality described exclusively in terms of the penis and the need for penetration; and (3) the melodramatic penis: descriptions that enshrine the penis as a revered icon of sexual pleasure for both men and women. A critical reading of an *Intimacy* article is offered under each theme in order to outline how it privileges both patriarchy and phallocentrism.

The study concludes by advocating for media texts to include alternative modes of male erotic pleasure as well as underscoring the role of primary care professionals with regard to promoting alternative forms of sex:

[*A*]cts that acknowledge the legitimacy and potential of non-genital erogenous zones for orgasm and/or pleasure. In doing so, patients are encouraged to explore sexual sensations and experiment with pleasurable feelings associated with the whole body.^14^

### Background

Sexuality has been a central concern of both the feminist movement and feminist scholarship.^2,3^ In particular, this concern has taken the form of a number of studies that have revealed that the scientific investigations and theories of sexological research have a gender bias and basis in which male-centred versions of sex are valorised.^2^ This is not just a peripheral idiosyncrasy but is argued to be the mainstay of the scientific basis of sexology which, from the early and late twentieth century, constructed a model of sexuality that purported to be both objective and scientific but, in fact, reflected and promoted the interests of patriarchy.^3,15^ In other words, the model of sexuality provided by scientific texts reflects patriarchal values by defining sex in male terms and, consequently, it controls and restricts women to specific expressions of sexuality.^15^

Yet, as suggested previously, science is not the only underlying contributing factor accountable for this viewpoint as the media is equally influential in producing normative notions of sex. These notions regulate current trends in sexual practices and result in the denial or denigration of sexual practices that depart from patriarchal norms.^2^ To this end, a number of feminist studies have also revealed that popular texts are reflective of patriarchal ideology.^2,16^ In exposing the patriarchal ideology, a number of shared themes are present and recur in popular texts, namely: male sexuality prioritised over female sexuality; and the favouring of penetrative sex over other sexual activities.^2^ In reviewing these themes, the reverberating finding is that popular texts articulate a set of sex and gender norms that serve patriarchy.^2^

This theorisation illustrates that there is a need for strengthening and expanding existing literature on sex – both scientific and popular – to offer an alternative version of female sexuality that does not imitate and/or epitomise patriarchal values. One attempt to do so is outlined by Rebecca Chalker, who advocates providing women with accurate and comprehensive information about their bodies and their sexuality.^17^ In terms of popular sex advice literature, this includes offering a broader definition of what constitutes sex and promoting a wider range of sexually pleasurable activities that are less reflective of a male-centred model of sexuality, instead exploring the specificities of female sexual pleasures.^8,17^ Explicit in this framework is that the possibility of an alternative and empowered female sexuality in heterosexual relationships requires male sexuality to depart from both patriarchal and phallocentric ideologies.^2^ Therefore, only by engaging with the promotion of alternative modes of male sexuality can an empowered female sexuality occur.^2,8^

Although *Intimacy* provides wide-ranging information on female health, well-being and sexual health issues, whilst also encouraging women to recognise themselves as sexual beings and to accept, assert and explore their sexual desires, the publication does not necessarily advocate sexual agency and individual autonomy for women.^8^ Instead, any identification of an ‘active’ or ‘empowered’ female sexuality is frequently oriented toward satisfying the pleasures and desires of a male-centred model of sex.^8^ Thus, whilst the magazine aims to empower women to take control of their sex life,^7^ this very attempt is hampered by the privileging and prioritisation of male sexual needs and desires.

## Research methods and design

### Sample

*Intimacy* is the English duplicate of the Afrikaans publication, *Intiem*. *Intimacy* was launched as a print publication but later adopted a solely web-based platform titled *INTIMACY4US*. The Afrikaans version, *Intiem*, continues in a print format. The sample consisted of the English bi-monthly issues of *Intimacy* from the July–September 2008 issue to the December 2009 – January 2010 issue, as well as the online articles accessed up until July 2011. *Intimacy*, as with most other magazines, contains a number of article types. These include, amongst others, feature articles, advertorials, advice columns, editorials, human interest, opinion articles, interviews, profiles and expert-authored texts. In order to narrow this down and produce a more focused analysis, the study consisted only of articles pertaining to the genre of an ‘informative content type’ (articles in which the focus on sexuality adopts the format of: what-to, how-to, when-to, why-to, etc.).

### Data analysis

Discourse analysis was utilised to identify the key themes that constitute *Intimacy*'s construction of male and female sexuality. The discourse analysis of the articles drew upon the method and guidelines suggested by Parker^18^ and the sample was coded into themes through a process of repeated reading. Through this strategy, interpretations and connections were developed, themes were refined and reworked and sub-themes were identified. To this end, each time a particular concept was identified in an article, all the other articles in the sample were re-read and re-examined in order to expand the concept into a specific theme or assign it to a sub-theme.

The analysis was informed by feminist theories of sexuality that examine the ways in which texts are associated with male-centred versions of sexual pleasure.^2,3,8,15^ However, this study makes no claim that this is the only possible reading of the articles in *Intimacy*. A fundamental feature of discourse analysis is that it acknowledges that there will always be the prospect of generating more appropriate or convincing interpretations.^19^ Moreover, alternative interpretations are to be expected by deploying a different theoretical framework or by sampling a different genre of articles from *Intimacy*. As indicated previously, *Intimacy* has many types of articles featured in each issue, from advertorials, to agony aunt columns, letters to the editor and even articles authored by experts in the field. Each respective article type holds the potential for a reading that departs from this study's findings.

## Results

As already indicated, three key themes regarding male sexuality have been identified in *Intimacy*. In the following section, the exploration of each theme will include a critical reading of one specific article in order to show how it reflects both patriarchal and phallocentric ideologies.

## Discussion

### Biological accounts of male sexuality

In the article, ‘20 ways a woman can superglue her marriage’,^20^ women are encouraged to be ‘actively involved in the bedroom’ and to improve their ‘sexual repertoire’.^20^ Such advice suggests that *Intimacy* advocates a degree of sexual agency for women. To elucidate further, the article encourages women to ‘[*b*]ecome comfortable with your sexuality and accept your body unconditionally. Know what stimulates you sexually, what you like and don’t like, and communicate this to your mate’.^20^ Such statements are salutary as they highlight the importance of sexual communication in constituting an empowered female sexuality. However, the potential to produce a fully-fledged sexual empowerment for women is limited by facets of male sexuality that are deemed to be non-negotiable and, consequently, waive the need for sexual communication. As will be argued below, these features of male sexuality are defined by *Intimacy* as being natural and biological facts and pivot primarily around the notion that men have an incessant need and desire for sex. For feminist theories of sexuality, such accounts are not objective or based on biological fact, but are instead patriarchal myths that are reinforced and perpetuated in popular, medical and sexological texts. Such myths maintain sexual privilege for men – that the ‘needs’ of the biological male sex drive dictate the sexual encounter.^3^ In turn, by upholding such myths, any consideration afforded the female partner or communication from her is silenced by stern warnings and rebuke.

To explore these points, a critical reading of the article ‘Do you refuse to have sex?’^21^ is offered. In the following quote, women are obliged to accept and satisfy their husband's biologically-entrenched drive and innate need for sex:

To explore these points, a critical reading of the article ‘Do you refuse to have sex?’^21^ is offered. In the following quote, women are obliged to accept and satisfy their husband's biologically-entrenched drive and innate need for sex:

Withholding sex from your husband deprives him of a deep-rooted need as basic as your own need to receive love from him regularly. He feels loved by you when you care enough about him to meet his physical needs and desire him enough to want physical relations.^21^

Here, women are urged to accept these features of male sexuality without discussion or reflection – to unconditionally accept his *biological* needs and wants. Equally problematic is the fact that the article cautions women against denying their husband this need and desire for sex as it will result in detrimental consequences for the marriage:

Your husband is mad about sex and thinks about it more than you do. Since the average man is more interested in sex than the average woman … he is more likely to: have strong sexual urges, take sexual risks – despite the consequences – be unfaithful or try commercial sex services. If you realise that merely the sight of your low neckline, your rounded bottom or the scent of your perfume can unleash this primitive instinct, you can’t help but realise that you should celebrate this ultimate attraction, as ignoring it could hold grave consequences for your marriage.^21^

In the above passage, male sexual satisfaction is acknowledged as an important means through which women can guarantee that men will remain unfailingly faithful to them. This places women in the role and responsibility of ensuring the ‘sexual upkeep’^8^ of men: women are urged to be willing and accepting of male sexual urges and constantly seek to satisfy them. In sum, female sexual communication is precluded when addressing the male sex drive. This holds significant repercussions for the extent to which women can be regarded as active and empowered in their sexuality. They may be able to communicate what they find sexually pleasing but are bound in having to submit to the dictates and prioritisation of the male sex drive.

### Phallocentric scripting of the sex act

Sex advice literature has been critiqued for denying the multiplicity of female sexuality – in particular, in reducing the female erogenous zones to only the vagina so as to comply with the coital imperative.^15^ However, in *Intimacy* a plethora of articles explore the multiple erogenous zones of a women's body – all of which have orgasmic potential. A number of these articles are especially written for men with a view to enabling them to sexually please their wives. These include: ‘22 things you need to know about your wife's body’^22^ that explores a number of non-genital erogenous zones that women find stimulating (including the neck, ears and navel); ‘Your wife's beautiful body …’,^23^ which lists a staggering 20 erogenous zones that hold the potential for female arousal and stimulation; and ‘Men only: How to be a man’^24^ that reiterates that ‘the vagina isn*’*t a woman*’*s only erogenous zone. Don*’*t forget about (amongst others) the ears, feet, neck, lips, thighs, eyelids, buttocks, nipples and breasts … and her brain*’*.^24^ Furthermore, a number of articles are related to coaching men in ways to ensure that their wives reach orgasm. Two examples are *‘*When the *“*Big O*”* plays hide and seek …*’*^25^ that outlines a list of steps for husbands to follow to ensure that their wives reach orgasm; and *‘*Men only: help her ride the waves!*’*,^26^ which stipulates the steps for men to follow in order to ensure that their wives experience multiple orgasms.

In contrast to the above, it will be argued that the sex advice written in *Intimacy* for women regarding their male partner*’*s sexuality is limited to the penis and penetrative sex. Anything departing from this is deemed foreplay. To elucidate further, in articles such as *‘*What he really wants in bed …*’*,^27^ which encourages women to explore their husband*’*s erotic zones, the accomplishment of this task is limited to exploring the genital erogenous zones.^27^ What is missing is the very discovery of a man*’*s non-genital erogenous zones. Furthermore, the article in question also impedes the enactment of potential non-phallic sex acts by displaying an inordinate focus and attention on the penis. What becomes comprehensible is that the penis becomes enshrined in copious consideration as an organ *par excellence.* A further compelling example of this ongoing relentless focus on the penis is evident in the article *‘*Don*’*t forget your mouth!*’*^28^ that encourages women to kiss, caress and love the penis.^28^

When women are encouraged to explore a man's non-genital erogenous zones, it is exclusively in terms of foreplay. In ‘Foreplay for those who’ve forgotten how…’,^29^ it is stated that:

[*w*]e are so inclined to pay most of our attention to the “typical” erogenous zones that we forget there are other just as sensual parts of the body. Decide to lavish attention on alternative zones, such as your mate's legs.^29^

Yet, this whole exercise in foreplay is only enacted to supposedly increase the ‘explosive force’^29^ of the subsequent penetrative sex act and the culminating orgasm. This aspect, in which foreplay is limited to increasing the ‘venting force’ of an orgasm during the sex act, is underscored later in the very same article:

Ask your mate to choose one non-erogenous zone on his body (for example his neck, ankles or tummy). Focus on this body part only, for the next 24 hours. Kiss that body part, tickle it, blow on it and cuddle it … be creative! Your goal should be to see how worked up you can get him in order to make your next session as explosive as possible!^29^

For male sexuality, foreplay is relegated solely to amplifying the pleasure linked to the final penetrative sex act. Foreplay is accorded no significance as a legitimate, genuine and noteworthy sexual act in its own right. To this end, foreplay is merely added to the standard phallocentric script: intercourse is still the main event and anything else is considered foreplay. Additionally, this standard scripting also sees that sex is construed as a linear process in which foreplay is followed by penetration.^30^ Consequently, both female and male sex acts are channelled into a limited form of expression in which sex takes on a particular linear pattern or sequence. Coitus remains the focus and endpoint in this sequence.^11,31^

Considering this section's findings, a number of critiques of *Intimacy’*s construction of male sexuality are perceptible. Although female sexuality is understood and heralded in its plurality and pervasive distribution of erogenous zones – all of which hold the possibilities for multiple orgasms – the converse is true for men. Male sexuality is described exclusively in terms of the penis and the need for penetration. For feminist theories of sexuality, this construct of male sexuality is reflective of phallocentric ideals. In this persistence of phallocentrism, vaginal intercourse is still deemed to be the sex act; everything else is relegated to foreplay. Thus, sex in such a framework lacks flexibility and non-penetrative, non-phallic possibilities.

### The melodramatic penis

In the previous sections, male sexuality was outlined as phallocentric (in relation to prizing coital sex) and patriarchal (female submission to the needs of the male sex drive). However, the phallocentrism presented in *Intimacy* also displays a significant departure from standard accounts addressed to men that enshrine penis size as of a high value for female pleasure and masculine ideals. For instance, in the article, *‘*Men only: how to be a man*’*,^24^ the size of the penis is proclaimed as being insignificant for mutual sexual pleasure:

Quality matters more than quantity: only the first 7,62 cm of the vagina benefits from stimulation, so you don*’*t need much more than that. When it comes to mutual sexual pleasure, penis length and girth mean nothing compared to the quality of foreplay, the sensitivity of touch, and the depth of intimacy in the relationship.^24^

In addition, the article continues to state that in regard to penis size that *‘*Nobody cares: it*’*s best to accept and appreciate what you have*’*.^24^ These quotes are helpful in offering a departure from standard phallocentric scripts that accord a larger penis size with the ability to provide a higher degree of female satisfaction. However, the advice written for women regarding the issue of penis size perpetuates a number of other phallocentric associations. These links are not explicitly apparent but are revealed in the contextualisation of sex, male sexuality and marital relations that following conventional/patriarchal gender norms and associations. To delineate further, in *‘*A small problem*’*,^32^ the article commences with the following statement:

On honeymoon, you wake up on the morning following a SECOND night without sex. When you peek under the sheets at your sleeping mate, you discover that he is … um … under-endowed. What next?^32^

Such a sensational proclamation is premised on a number of hegemonic accounts of male sexuality *–* for example, that normal men are sexually potent and incessantly require sex. Yet, even more perturbing, is that the statement is reductive: the lack of sex is based on the man*’*s small penis; not on the man in question *–* his values, desires, psychology and interpersonal factors that are reflected in his sexual needs, frequency and responsiveness. Although the quote may be argued to be qualified in a more grounded and sensible account in later paragraphs, it is still based on an unwavering persistence of patriarchal ideals of male sexuality evident in the use of the term *‘*virility*’*:

If you suspect that your husband is a little self-conscious about the *‘*tools*’* under his belt, share the following facts with him … the length and width of a man*’*s penis has nothing to do with his virility.^32^

In this account, the use of the term *‘*virility*’* is a propagation of patriarchal definition and values. Furthermore, the quote also reveals marital relations that are ascribed according to gendered divisions and roles. Women are provided with guidance in order to support their husbands. As such, the patriarchal description of women as a *‘*helper*’* for her man includes offering support to shore up and reassure him against any feelings of inadequacy or inferiority that he may have regarding his penis size. This is unmistakably noticeable in the following quote:

If hubby feels intimidated by what he has seen in blue movies, comfort him with the information that only men with unusually large penises are used as actors and even then, their *‘*equipment*’* is made to look bigger using make-up and special photographic effects.^32^

The advice written for women regarding penis size can be discerned to reflect patriarchal views: the continuation of essentialist accounts of male sexuality (that men have an unrelentingly high sex drive and sexual responsiveness that is without a bearing on individual/interpersonal aspects and external factors); patriarchal terminology (male virility); and marital relations (the wife as the support and comfort for her husband). Yet, these very accounts do not construct the penis in terms of either a phallic spectacle (big, powerful and impressive) or as pitiful and shameful stemming from its disjuncture from the phallus (small and weak). As such, it departs from the polarity which structures the dominant discourse of the penis in the West: the dichotomy of phallic versus non-phallic.^33^ Rather, the penis reflects a third category, termed the *‘*melodramatic penis*’*.^33^ Peter Lehman coins the term *‘*melodramatic penis*’* to account for the discourses of the penis that do not polarise the penis as phallic/non-phallic but continue to affirm the spectacular importance of the penis.^33^

The discourse of the melodramatic penis includes a number of characteristics which will be explored further. Firstly, the melodramatic penis can be read in a positive manner as avoiding the simple structuring dichotomies of the large, awesome phallic spectacle versus its abject antithesis. In this sense, the penis is removed from either the ideal phallic spectacle or the ridicule of the ineptitudes of real penises. Secondly, although not presented within a dichotomy, the discourse of the melodramatic penis persists in defining the penis in terms of monumental importance – the penis is seen to be manifest in connotations associated with male sexuality, health and well-being that continue to block a penis from merely being a penis.^33^ In this regard, the penis is no longer fixed in a dichotomy of phallic and non-phallic but it is still marked as connoting extraordinary meaning.^33^ Thus, the discourse of the melodramatic penis challenges conventional representations, yet it remains a troubled site of representation as it continues to frame the penis in awe and mystique.^33^ In other words, the melodramatic penis defies traditional dichotomies (phallic versus non-phallic) whilst maintaining and securing its importance in the sex script which continues to preclude the penis from being just a penis.^33^

To return to the article in question, the discourse of the melodramatic penis is apparent in the advice written for women, in which there is an appeal to the emotions of women to provide comfort and support for men in order to quell any lack of penis-confidence that they may have. Additionally, rather than merely being a body part, the penis is framed in terms of virility. It is also treated as a default conjecture in assessing the lack of male desire for sex and sexual responsiveness. Yet, what most epitomises the melodrama of the penis is the lack of the very identification of it as penis, an organ and not an extension of masculinity and gender stereotypes: an organ rather than a symbol that is embedded and assigned meaning in terms of virility; an organ rather than a motif of anxiety and feelings of insecurity; and an organ that is inconsequential to mutual sexual pleasure. Even in the closing remarks of the article in question, the melodrama of the penis is present:

So, if your wedding night is coming up and you are wondering what*’*s in your lucky dip, don*’*t be anxious. You probably won*’*t get a good look at your husband*’*s penis on the first night *–* but, hopefully, you will find out what this wonderful apparatus can do for you! If he is skilful, there is little chance that you will ever guess its true size.^32^

The use of the term ‘lucky dip’ continues to mask the penis from being exactly what it is. In perpetuating such descriptions, the penis becomes a metaphor for pleasure and satisfaction; as tools, equipment and apparatuses for both female and male sexuality. Such metaphors persist in framing pleasure as a quality or capability of the penis. This is at the expense of outlining accounts of male and female sexuality in terms of mutual pleasure derived not from the penis or penetration but from contact, connection and the closeness of a sexual/relationship bond.

In sum, the discourse of the melodramatic penis is a departure from the dichotomist construction of the ideal, potent phallus set against the fallibility inherent in attempting to live up to this ideal. However, it still enshrines the penis with astounding importance in terms of offering pleasure for both men and women. Thus, even though the phallic qualities of large penis size are not present in the article, the discourses of the melodramatic penis continue to reinforce phallocentric descriptions of sex (the penis as an organ of pleasure for both sexes) and patriarchal relations (woman as a man's aid, offering him both support and reassurance in regard to penis size).^9^

## Conclusion

The study has revealed that *Intimacy*'s aim to ‘empower you as its reader and give you permission to take control of your sex life’,^34^ is, at best, only a pseudo-empowerment for women in heterosexual relations.^8^ It can only ever promote an illusory sense of female control and pleasure as it persists in defining male sexuality according to patriarchal standards. The patriarchal underpinning of male sexuality in *Intimacy* has been revealed to delimit the sexual act, female sexuality and men to predefined potentials and gender relations: restriction of male sexual expression to the erect penis; notions of ‘real’ sex as penile-vaginal penetration (at the expense of diverse erotic experiences derived from non-genital erogenous zones); biological accounts of the male sex drive (that negate acts of communication and negotiation); the relegation of any sexual act that departs from coitus to foreplay (and thus of secondary importance); and the continual description of the penis as a revered icon of sexual pleasure for both men and women.

To offer a departure from the above findings, constructions of male sexuality require the inclusion of alternative modes of male erotic pleasure. This requires media texts and primary care professionals to encourage men to explore and also to experiment with pleasurable feelings associated with non-genital erogenous zones of the body.^2^ Accordingly, in the case of primary care professionals, their role is to ‘reintroduce sex [*within*] … as wide a definition possible’^35^ in order to propose an expansive view of male sexuality that affirms pleasure over the whole body. Such transformation is not to expose the inadequacies and limitations of male sexuality, but to disestablish the dominance of patriarchal and phallocentric versions of sex.^2^ In doing so, it holds the potential of changing the sexual act via the exploration of non-phallicised versions of sex and sexuality, as well as empowering female and male sexuality to unlimited potential within a relationship based on mutual respect and sexual communication.
